# Enhanced oxygen availability and preserved aggregative function in platelet concentrates stored at reduced platelet concentration

**DOI:** 10.1111/trf.18101

**Published:** 2024-12-14

**Authors:** Jamie Nash, Dean Pym, A. Davies, Christine Saunders, Chloe George, J. O. Williams, O. Y. Grinberg, Philip E. James

**Affiliations:** ^1^ Center for Cardiovascular Health and Ageing Cardiff Metropolitan University Cardiff UK; ^2^ Component Development and Research Laboratory Welsh Blood Service NHS Wales UK; ^3^ Dartmouth Medical School Hanover New Hampshire USA

**Keywords:** blood component preparations, platelet transfusion, transfusion practices (adult)

## Abstract

**Background:**

Storage of platelets as platelet concentrates for transfusion is limited to 7 days in the United Kingdom due to deleterious effects on platelet quality and function that occur over time. Oxygen (O_2_) availability and sufficient gaseous exchange are known to be essential in maintaining the viability and function of platelets stored for transfusion. Despite this, there is a paucity of studies undertaking direct measures of O_2_ and optimization of conditions throughout storage. We address this and modulate the storage conditions to improve platelet quality and function.

**Study Design and Methods:**

Electron paramagnetic resonance oximetry was implemented to directly measure the [O_2_] experienced by stored platelet concentrates and the O_2_ consumption rate under standard blood banking conditions. From these direct measures the mathematical modeling was then applied to predict the main parameters contributing to effective O_2_ distribution throughout the unit.

**Results:**

This study demonstrates reducing the storage [O_2_] to reflect near physiological levels significantly alters O_2_ distribution within the unit and negatively impacts platelet functionality and quality, and therefore is not a viable storage option.

**Discussion:**

We show the reduction of platelet concentration within a unit improves O_2_ availability and pH, promotes a more uniform distribution of O_2_ throughout prolonged storage, and maintains platelet agonist‐induced aggregation comparable to 100% platelet concentration. This may be a viable option and could potentially lead to reduced donor demand.

## 1 INTRODUCTION

Oxygen (O_2_) availability and sufficient gaseous exchange are essential in maintaining the metabolism, viability, and function of platelets (PLTs) stored for transfusion.^1^, ^2^, ^3^, ^4^ With ~15,000–20,000 platelet concentrates (PC) supplied by the Blood Transfusion Service annually in Wales (Welsh Blood Service, WBS), the demand for PLT donations remains high due to the deleterious changes that occur to PLTs from the point of blood withdrawal to the time of transfusion, limiting PC storage to a maximum shelf life of 7 days. A series of studies conducted by Murphy et al. reported O_2_ availability and glucose supply to be essential for maintaining PC quality and function, laying the foundation for prolonging PC storage from 3–5 days to 5–7 days.^1^, ^4^, ^5^, ^6^ Despite the improved gas permeability of modern storage containers, few studies have further investigated whether oxygen concentration [O_2_] could be further optimized to improve PLT quality during storage.

The literature describes the average pO_2_ range in peripheral tissues as ~8–40 mmHg (10‐ to 54‐μM [O_2_]).^7^ Clearly, this will depend on the tissue and composition of cells, the O_2_ requirement, and the O_2_ source. Over the last decade, the use of optimized (often‐lower) perfused O_2_ has been applied to cell culture systems to mimic in vivo conditions—termed “physioxia” or “normoxia”—to promote favorable growth conditions, and maintenance of cell viability and function. Lowering [O_2_] has been successfully applied to culture of mouse embryonic fibroblasts,^8^ hematopoietic stem cells,^9^ and endothelial cells (3%–5% O_2_), resulting in increased growth,^10^ decreased Bcl2 expression,^11^ and leading to a distinct normoxia‐induced phenotype.^12^

Given that arterial pO_2_ is typically in the range of ~80–100 mmHg (~107–135 μM [O_2_]), whereas venous pO_2_ ~30–50 mmHg (~40–67 μM [O_2_]),^13^ it is not surprising that optimization of [O_2_] conditions for storage of blood components has also been investigated. A major concern with red blood cell (RBC) storage is hemoglobin oxidation^14^, ^15^ and reactive O_2_ species (ROS) generation,^16^ resulting in depleted ATP, loss of cell surface area, and eventual hemolysis.^17^ RBCs use glycolytic pathways for metabolism; therefore, relatively high O_2_ is not a requirement for RBC survival.^18^ RBCs stored in a hypoxic environment discourage hemoglobin oxidation, and produce less ROS, resulting in a decrease in RBC damage.^18^ The investigations concluded that improved energy generation and decreased oxidative stress in RBCs resulted in enhanced posttransfusion recovery in patients,^19^ confirming lower O_2_ storage conditions can promote the viability and function of blood products.

To determine if PC storage can be further optimized, conditions predicted to influence PLT O_2_ availability, including the ambient storage [O_2_] and the platelet concentration [PLT], were modified experimentally. Electron paramagnetic resonance (EPR) oximetry was implemented to measure the [O_2_] experienced by PLTs within the storage container and the O_2_ consumption rate of PCs stored under standard blood banking conditions. From these direct measures, mathematical modeling was then applied to predict the main parameters contributing to effective O_2_ distribution throughout the PC and investigate whether storage guidelines for PC could be optimized to allow for improved PLT quality or lifetime.

## 2 METHODS

### 2.1 Preparation and storage of platelet concentrates (PC)

Blood was donated by healthy individuals according to approved Welsh Blood Service (WBS) standard operating procedures and used under IRAS approval number 251196.^20^, ^21^ Buffy coats (BC) obtained from whole blood donations were held overnight at 22°C ± 2°C. Informed consent was obtained from all donors to allow sample use in transfusion research, according to WBS protocol. Ethical opinion was approved by the Cardiff Metropolitan University Ethics Committee (ref#PGR‐1301). Four ABO‐specific BC were sterile connected and pooled using 250 mL of SSP+ platelet additive solution (PAS) (Macopharma, Moureaux, France) as a suspension medium, providing a PAS to plasma ratio of ~65:35. The BC and PAS pool were then centrifuged at 500*g* for 5 min, followed by separation of the PC from residual red and white cells using a blood component separator (CompoMat G5+, Fresenius Kabi, Germany). Leukoreduction occurred at the time of separation through a reduction filter (Autostop; Haemonetics, Boston, MA). PCs were pooled and split before storage. To produce a PC with lower [PLT] 50% of the PC was removed as required, before being replaced with an equal volume of 65:35 PAS plasma solution. A sample was taken for a PLT count on a hematology analyzer to confirm the reduced [PLT] was correct for the given dilution.

PC was stored at 22°C ± 2°C with constant 60 Hz agitation with a horizontal displacement of ±2.5 cm, either in ambient air (21% [O_2_]), or at 10% or 5% [O_2_] in a modified In VivO_2_ chamber (Ruskin Ltd). PC were given a 24 h stabilization period post‐harvest to establish equilibrium at the set [O_2_] conditions. A PC containing only PAS was utilized to determine the effect of the container and solubility of O_2_ in the absence of PLTs. PCs were spiked with a sampling port and aseptically sampled on days 2, 4, 6, 8, and 10 of storage, at no point exceeding a total sampling volume >10% of PC in accordance with guidelines.

### 2.2 Electron paramagnetic resonance O_2_ oximetry

#### 2.2.1 Calibration and [O_2_ ]

EPR oximetry was performed using Per Deuterated, N^15^ Tempo (0.2 mM; PDT) in PAS drawn into a gas permeable Teflon tube and inserted into a quartz capillary tube open at both ends. Calibration was undertaken with either pure Nitrogen (0% O_2_) or air (21% O_2_) and the spectral linewidth was taken to reflect [O_2_]_nitr_ and [O_2_]_air_, respectively. This technique is well established for assessing [O_2_] in cell samples and relies on the fact that the EPR spectral linewidth of PDT shows a near‐linear relationship with [O_2_] in this range (see Figure S1). [O_2_] measured in the experimental sample ([O_2_]_sample_) can then be calculated according to Equation (1).

(1) $$\;\frac{{\left[O_{2}\right]}_{\text{sample}}-{\left[O_{2}\right]}_{\text{nitr}}}{{\left[O_{2}\right]}_{\mathrm{air}}-{\left[O_{2}\right]}_{\text{nitr}}\;}\times 210\mathrm{\mu M}.$$

### 2.3 Oxygen consumption rate (OCR)

PC sample (typically 1 × 10^8^ PLTs) with ^15^N PDT (0.2 mM) was drawn into a glass capillary tube, then sealed at both ends using Critoseal to create a closed chamber. EPR spectra were recorded at 1 min intervals for 30 min, or until the sample linewidth = [O_2_]_nitr_ to generate a linear plot of spectral linewidth against time. The rate of linewidth change (see Figure S2). was converted to [O_2_] using Equation (1) above and an OCR calculated as the change in [O_2_] per volume of cells over time. The initial linewidth was taken to reflect the [O_2_] directly inside the bag. Measures were first recorded at day 2 for PC and a PAS only sample was taken to reflect the maximal O_2_ capacity of the storage container in the absence of PLTs. To compare OCR with freshly isolated PLTs, PLT rich plasma (PRP) was isolated from whole blood collected in a citrate vacutainer and centrifuged at 250*g* for 10 min at room temperature. Oxygen sensitive ^15^N PDT was added to the PRP sample and the OCR experiment conducted as for PC above. OCR are expressed corrected for [PLT] unless otherwise stated.

## 3 PLATELET CONCENTRATE FUNCTION AND QUALITY

Multiple electrode aggregometry employing Multiplate (Roche Diagnostics Ltd., Switzerland) measured electrical impedance across electrodes to reflect PLT aggregation in real time.^22^ PC (200 μL) were diluted 2:1 with SSP+™ PAS. Diluted PCs were further diluted to the manufacturer's instructions of 1:1 with 0.9% NaCl when testing with Ristocetin or 0.9% NaCl with 3 mM CaCl_2_ when testing with Thrombin Receptor Activator Peptide 6 (TRAP‐6) and preheated to 37°C. A Teflon coated stirring bar kept the sample uniformly mixed. PLT aggregation was initiated by addition of either Ristocetin (50 μL, 0.77 mg/mL final concentration) or TRAP‐6 (20 μL, 32 μM final concentration) (Roche Diagnostics Ltd., Switzerland). Adenosine diphosphate (ADP) was not used due to the phenomena of ADP desensitization in fresh buffy‐coat‐derived PCs.^23^ Electrical impedance was measured over 6 min and the results expressed as area under the curve (AUC).

An important quality control (QC) measure of PC commonly applied worldwide is pH.^24^ pH should not drop below a lower acceptable threshold of 6.4 at 22°C. Samples were taken aseptically from the PC and sampled on a HORIBA LAQUAtwin pH meter, which was calibrated before use.

### 3.1 Oxygen modeling

**FIGURE 1:**
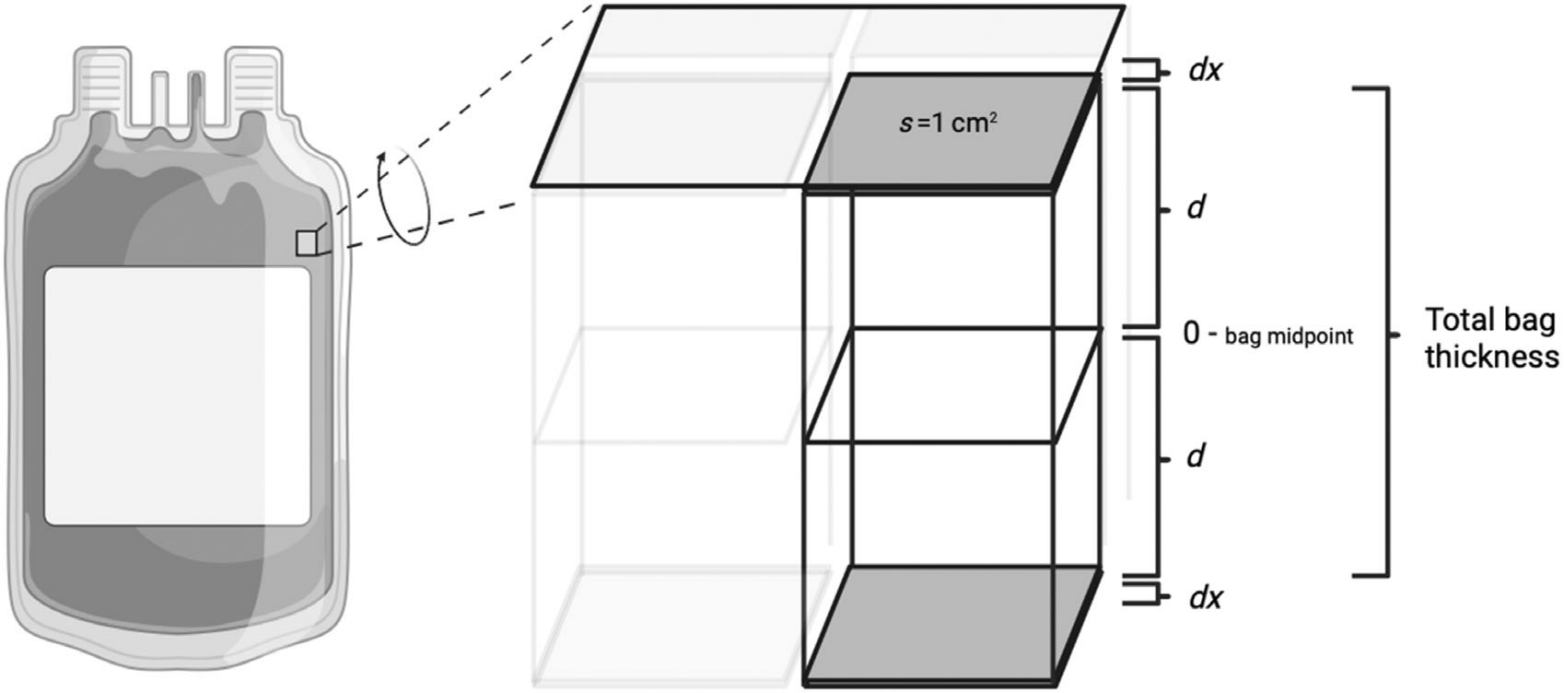
Diagram to reflect the conceptual basis of the O_2_ modeling. Gray layer = the plastic film of the bag, *dx* = 0.035 cm is the film thickness, *s* = 1 cm^2^ is the surface area, *d* = 0.53 cm is half the width of the platelet concentrate unit.

The influx of O_2_ through the bag surface was first modeled using Fick's principle (assuming unidirectional steady state diffusion), where *J* = oxygen flux (*J*_in_), *D* = diffusion coefficient of the bag film (9.84 × 10^−8^ cm/s), [O_2_] = O_2_ concentration, *s* = surface area (1 cm^2^), and *dx* = film thickness (cm) (Equation (3)). For simplicity, the bag is divided into a series of individual uniform parallelepipeds acting through an area of 1 cm^2^, through a length equal to half thickness of a full PC bag (Figure 1). For this model the distribution of PLTs was assumed to be homogenous, with uniform PLT number within each repeating unit. Since the model is based on the OCR per parallelepiped, the local PLT distribution will likely have little effect on the summative O_2_ consumption. The total OCR per parallelepiped was calculated using Equation (4), where *d* is half of the bag thickness.

(2) $$J=D\;\frac{\nabla \left[O2\right]}{\mathrm{dx}*s},$$

(3) TotalOCR=MeasuredOCR*d.

It was of interest to model how closely the [O_2_] measured experimentally fits a homogenous distribution. A one‐dimensional approach was used due to the symmetry of the task. A one‐dimensional steady‐state diffusion equation of oxygen concentration [O_2_] inside the bag can be described by Equation (4).

(4) $$\frac{d^{2}\left[O_{2}\right]}{{\mathrm{dx}}^{2}}-\frac{\mathrm{OCR}}{D}=0,$$

where, *d* ^2^[O_2_]/*d*𝑥^2^ is a second derivative of oxygen concentration, *D* is the oxygen diffusion coefficient in the media (assumed equal to water at *D* = 2.42 × 10^−5^ cm^2^/s), OCR is oxygen consumption rate. The solution of Equation (4) presents the distribution of [O_2_] inside the parallelepipeds and can be expressed by Equation (5).

(5) $$\left[O_{2}\right]=\frac{\mathrm{OCR}}{2*D}*x^{2}+C_{1}x+C_{2},$$

where, *C*_1_ and *C*_2_ are constants. [O_2_] in the center (where *x* = 0) achieves minimum, so the first derivative *d*[O_2_]/*dx* = 0, and therefore *C*_1_ = 0. Integration of Equation (5) resolves constant *C*_2_ and allows us to model the average oxygen concentration <[O_2_]> in the bag (Equation (6)).

(6) $$C_{2}=<\left[O_{2}\right]>-\frac{\mathrm{OCR}}{D*6}d^{2}.$$

Finally, the distribution of [O_2_] can then be described by Equation (7), where [O_2_] represents oxygen concentration at a given position within the PC, “OCR” represents oxygen consumption rate, *D* represents the diffusion coefficient, “*x*” represents the distance from the center of the bag (excluding film thickness), “<[O_2_]>” represents the O_2_ measured, and “*d*” represents half the width of the PC. Based on this, representative plots of [O_2_] distribution according to distance traversing the bag were produced using R studios for presentation in this manuscript.

(7) $$O_{2}=\frac{\mathrm{OCR}}{2*D}*x^{2}+<\left[O_{2}\right]>-\frac{\mathrm{OCR}}{D*6}d^{2}.$$

## 4 STATISTICAL ANALYSIS

Data analysis was performed using GraphPad Prism 9 software (San Diego). A Shapiro–Wilk test was used to determine that all data were normally distributed before statistical testing. Statistical significance was inferred by a paired *T*‐test when comparing the means of two paired units, or a one‐way ANOVA with Tukey's post hoc test when comparing the means of more than two independent groups. A two‐way ANOVA, followed by a Bonferroni post hoc test was used to compare means of more than three independent variables, when accounting for the levels of two categorical variables. A *p* value of <.05 was considered statistically significant.

## 5 RESULTS

### 5.1 Effect of reduced external O_2_ on direct O_2_ concentration and consumption within PC

**FIGURE 2:**
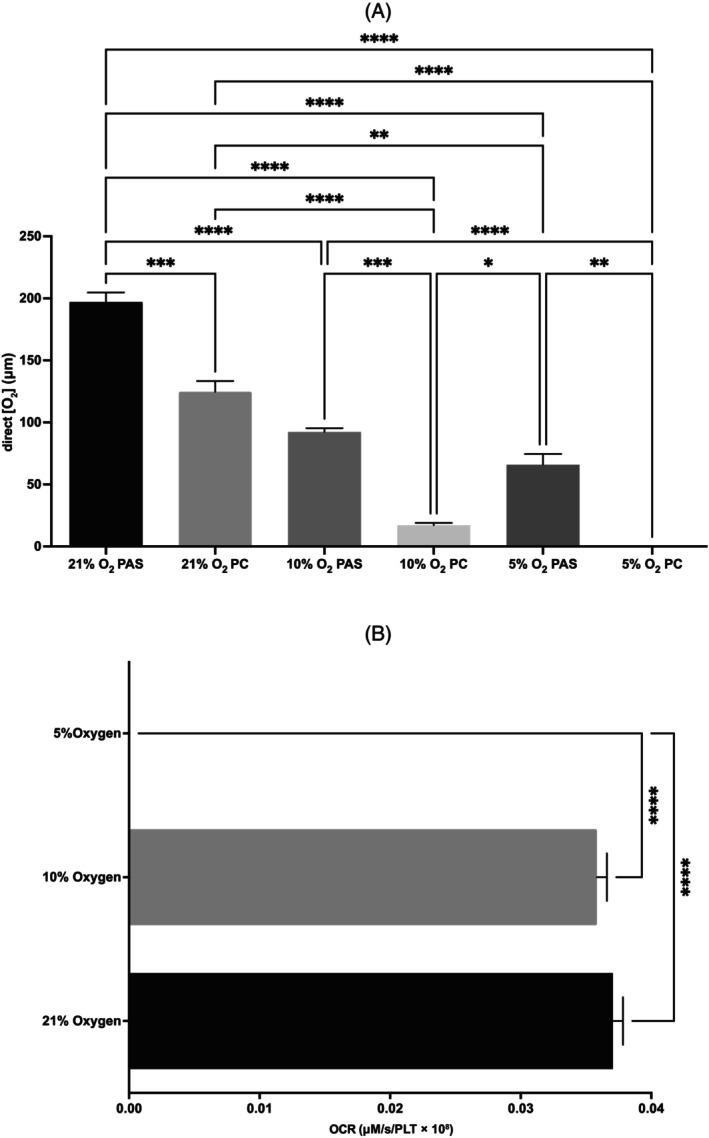
The effect of a reduced external [O_2_] on direct [O_2_] and oxygen consumption rate (OCR) in platelet concentrates (PCs). (A) Direct [O_2_] within PC units stored on day 2 at different external [O_2_]. (B) OCR under corresponding conditions. Error bars denote SEM. Significance was assessed by one‐way ANOVA followed by a Tukey's test. Graphs denoted as follows: ns = no significance, * = *p* < .05, ** = *p* < .01, *** = *p* < .001, **** = *p* < .0001, *n* = 3 (*n* = 6 control).

[O_2_] was measured in PAS only and PC samples on day 2 of storage to assess the [O_2_] in the bag in the absence and in the presence of PLTs, respectively, at the beginning of the investigation (Figure 2A). With the ambient [O_2_] at 21%, 10%, and 5%, the direct [O_2_] measurement within PC was 124.7 ± 24.49 μM, 17.16 ± 3.14 μM (*p* < .0001), and 0 ± 0 μM, respectively. [O_2_] was significantly higher in PAS only compared with PC at 21% [O_2_] (*p* < .01) and 10% [O_2_] (*p* < .001). The [O_2_] was significantly higher in 21% [O_2_] PAS only compared with all other conditions irrespective of content (*p* < .0001), with [O_2_] measured in PAS only samples reflecting the external [O_2_] very closely under each condition.

OCR was not significantly different between 21% [O_2_] and 10% [O_2_] in PC; however, at 5% [O_2_] the measured OCR decreased to zero (*p* < .0001, Figure 2B). To maintain a measured [O_2_] level within the bag = 0, the OCR must be equal to the influx of O_2_ into the PC (*J*_in_). Thus, assuming total OCR = *J*_in_ (Table 1), the OCR would be limited to 0.0297 μM/s/PLT × 10^8^. *J*_in_ is the same at 21% [O_2_] and 10% [O_2_] but decreases significantly at 5% [O_2_] (*p* < 0.05). For comparison, the OCR of PLTs within a fresh PRP sample (with minimal processing) was 0.031 ± 0.004 μM/s/PLT × 10^8^.

**TABLE 1:** The effect of variation of external [O_2_] and [PLT] on O_2_ influx and total oxygen consumption rate.

External [O_2_]	[PLT]	*J*_in_ (molec/cm^2^/s)	Total OCR (molec/cm^2^/s)
21% O_2_	100%	13.4 ± 5.07	11.7 ± 0.546
	50%	7.34 ± 4.86	5.09 ± 1.6
10% O_2_	100%	15.2 ± 0.57	11.7 ± 0.424
5% O_2_	100%	9.18 ± 0.0	≥9.18

*Note*: The modeled O_2_ influx (*J*_in_) and total OCR given using Equations (3) and (4), respectively. Values were reported as mean values ×10^10^ with ± representing SD.

### 5.2 Effect of reduced [PLT] on [O_2_ ] and OCR in PC

**FIGURE 3:**
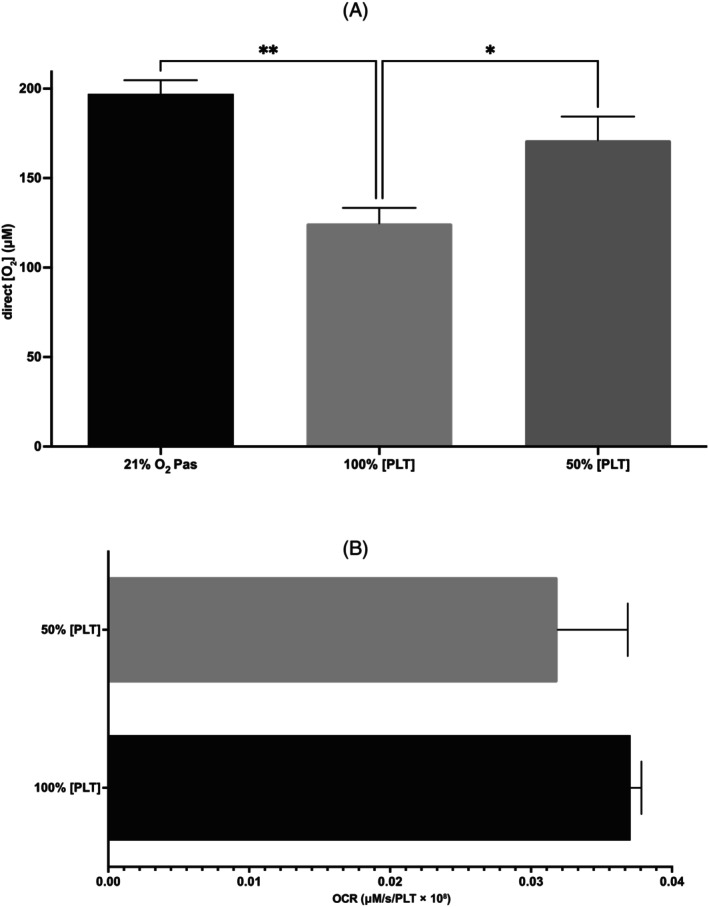
The effect of a reduced [PLT] on direct O_2_ and oxygen consumption rate (OCR) in platelet concentrates (PCs). (A) Direct [O_2_] measurement within PC units on day 2 storage at 100% and 50% [PLT]. (B) OCR at 100% and 50% [PLT], when normalized for PLT number. Error bars denote SEM. Significance was assessed by one‐way ANOVA followed by a Tukey's test (A) or a paired *T* test (B). Graphs denoted as follows: * = *p* < .05, ** = *p* < .01, *n* = 4.

As PLT concentration was reduced from 100% to 50%, [O_2_] measured on day 2 storage significantly increased from 124.7 ± 22.9 μM, to 171.1 ± 22.9 μM (*p* < .05, Figure 3A). There was a significantly lower [O_2_] in PC at 21% compared with PAS only at 21% [O_2_] (*p* < .01).

We observed no significant difference in OCR as PLT concentration was reduced from 100% to 50%, when normalized for PLT count (Figure 3B). Despite this, there is a significant reduction in the total O_2_ consumed per unit volume at 50% [PLT] compared with the 100% [PLT] (*p* < .05). Correspondingly, *J*_in_ also significantly decreases with decreased [PLT] (*p* < .05) (Table 1).

### 5.3 Effect of reduced external [O_2_ ] on O_2_ distribution within PC

**FIGURE 4:**
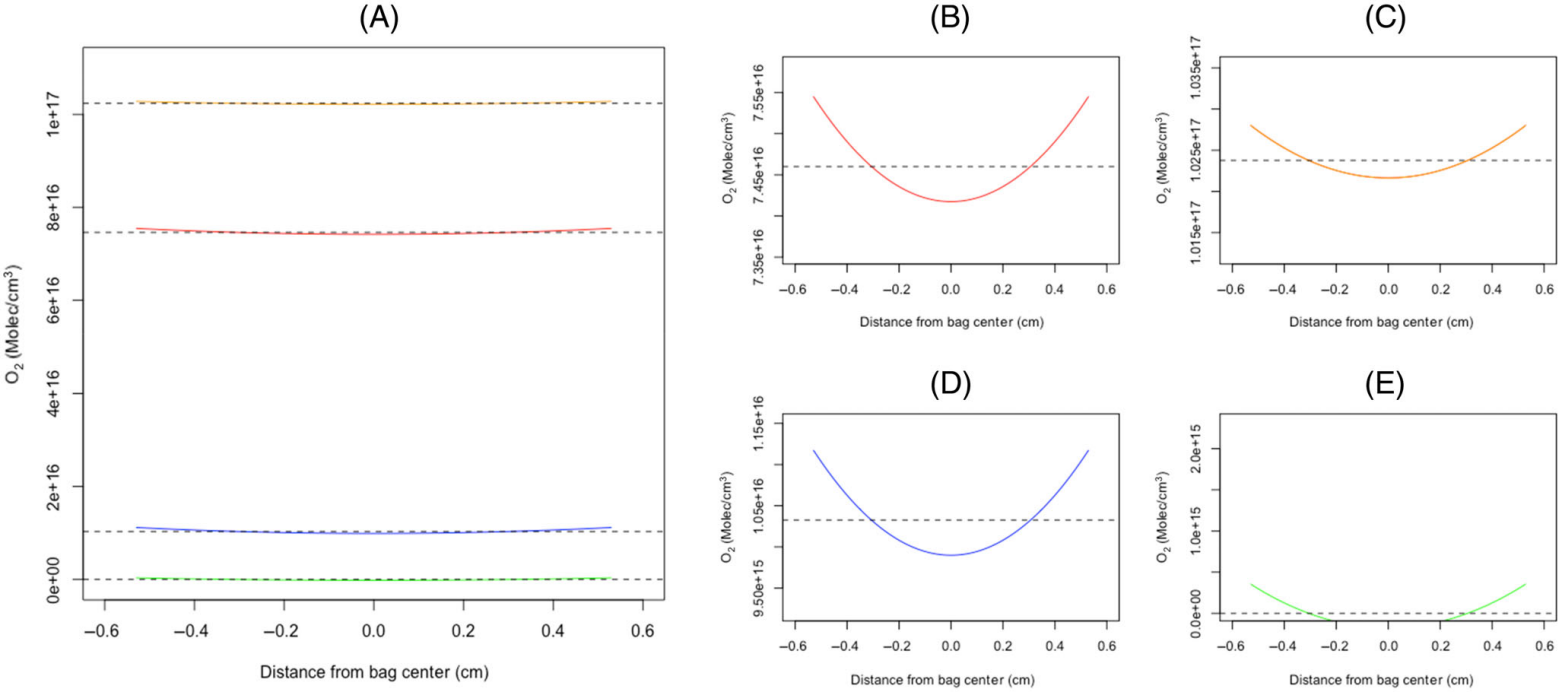
Modeled O_2_ distribution within platelet concentrate (PC) units incubated at different external [O_2_]. Modeling was undertaken using Equation (3). The *X* axis describes the distance from the bag midpoint, with 0 representing the bag center, and 0.53 representing the bag surface. The *Y* axis shows the oxygen concentration. The part figure (A) compares the O_2_ distribution for 100% [PLT] stored at 21%, 10%, and 5% external [O_2_], and 50% [PLT] stored at 21% [O_2_]. The part figure (B) is expanded *Y* axis showing the O_2_ distribution for 100% [PLT] at 21% [O_2_], (C) shows the O_2_ distribution for 50% [PLT] stored at 21% [O_2_], (D) shows the O_2_ distribution for 10% [O_2_] with 100% [PLT], and (E) shows the O_2_ distribution for 5% [O_2_] with 100% [PLT]. The curve highlighted in red, or orange correspond to the predicted internal O_2_ distribution for PCs stored at 100% and 50% [PLT], respectively, stored at an external O_2_ of 21% [O_2_] (panels B and C), the blue curve represents the internal O_2_ distribution predicted at an external [O_2_] of 10% (panel D) and the green curve represents the internal O_2_ distribution predicted with the external [O_2_] at 5% (panel E). The dashed lines denote the experimental average [O_2_] measured by electron paramagnetic resonance at each condition. [Color figure can be viewed at wileyonlinelibrary.com]

O_2_ distribution within the PC was predicted from the direct measures and Equation (7) (Figure 4). The distribution of [O_2_] across PC stored at 21% [O_2_] and 10% [O_2_] deviated from the average measured [O_2_] at the inner surface and the midpoint by 8.51 × 10^14^ molec/cm^2^ and 4.26 × 10^14^ molec/cm^2^, respectively (Figure 4B,D, respectively). Interestingly, this only reflects a relative O_2_ distribution of 0.57% and 1.14% from the average measured [O_2_], compared with 4.2% and 8.2% at 10% [O_2_]. Despite this, at 21% and 10% [O_2_], there is no significant difference between the [O_2_] at the inner bag surface compared with the bag midpoint (21% [O_2_]: *p* > .05, 10% [O_2_]: *p* > .05).

PC stored at 5% [O_2_] showed a more homogenous distribution compared with the PCs stored at 10% [O_2_] and 21% [O_2_], largely because the OCR was apparently reduced (0.0297 μM/s/PLT × 10^8^ compared with 0.0367 μM/s/PLT × 10^8^). Moreover, using the modeled OCR for 5% [O_2_], and the [O_2_] at the surface of the bag = 3.55 × 10^14^ molec/cm^2^, our model predicts >60% of the PC to be in complete anoxia at any given time when stored at 5% [O_2_] (see Figure 4E where the values of the predicted [O_2_] fall lower than zero).

### 5.4 Effect of reduced [PLT] on O_2_ distribution within PC

The predicted [O_2_] distribution in PCs stored at 50% [PLT] deviates less from a homogenous distribution compared with PCs stored at 100% [PLT] (Figure 4B,C, respectively). Comparing the inner edge of the bag to the midpoint, PCs stored at 100% [PLT] deviate from homogeneous [O_2_] by 8.51 × 10^14^ molec/cm^2^ (1.14%) and 4.26 × 10^14^ molec/cm^2^ (0.57%), respectively, while PCs stored at 50% [PLT] deviate from homogeny by 4.26 × 10^14^ molec/cm^2^ (0.41%) and 2.13 × 10^14^ molec/cm^2^ (0.2%), respectively. At both stored [PLT]s, there was no significant difference between the [O_2_] at the inner bag surface compared with the bag midpoint (*p* > .05).

### 5.5 Effect of reduced [PLT] on [O_2_ ] and OCR over PC storage

**FIGURE 5:**
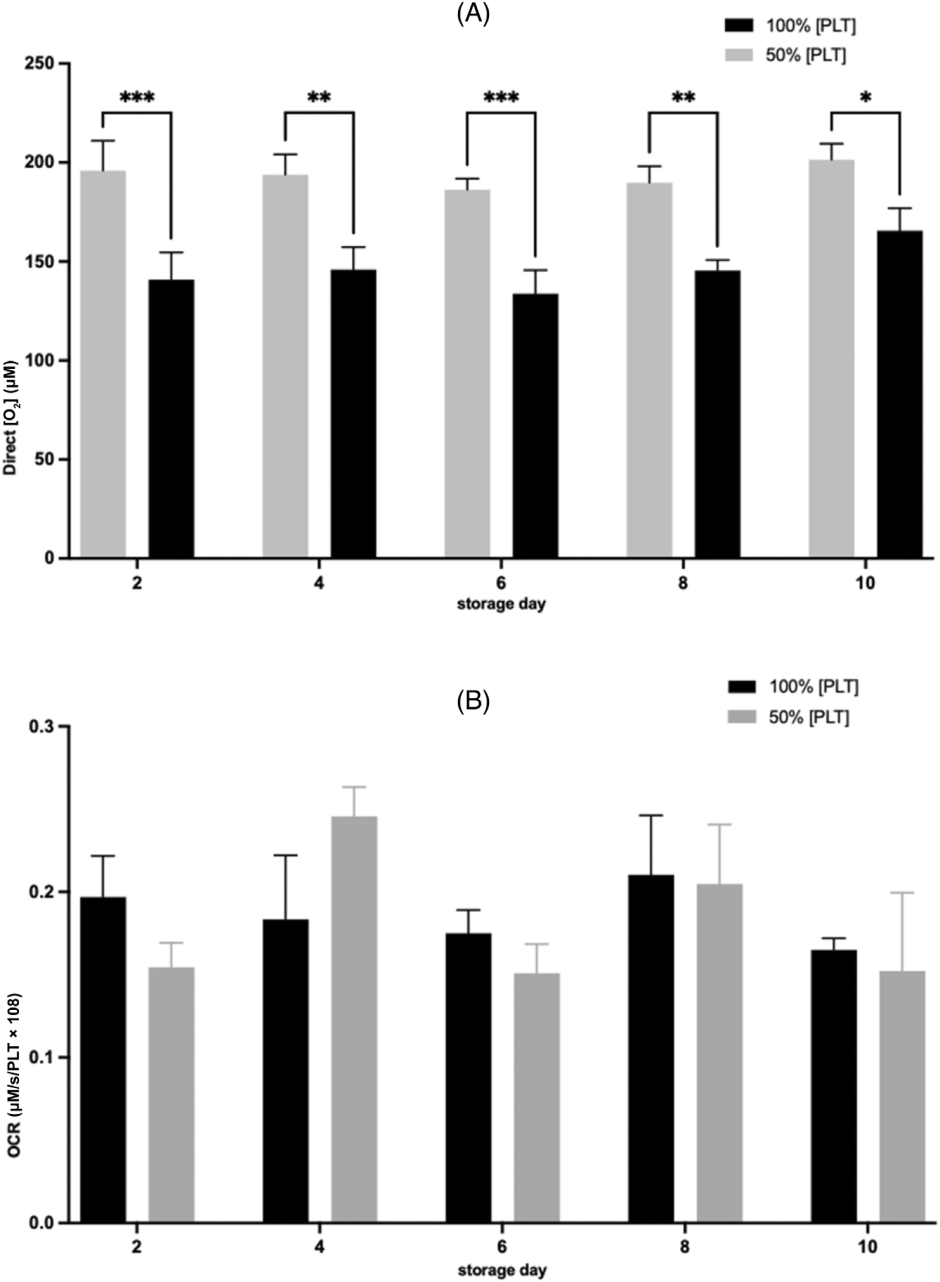
The effect of a reduced [PLT] on direct O_2_ and oxygen consumption rate (OCR) in platelet concentrates (PCs) over 10 days of storage. (A) Direct [O_2_] measurement within PC units at 100% and 50% [PLT]. (B) OCR at 100% and 50 [PLT], when normalized for platelet number. Error bars denote SEM. Significance was assessed by two‐way ANOVA following by a Bonferroni test and graphs are denoted as follows: * = *p* < .05, ** = *p* < .01, *** = *p* < .001, *n* = 4.

There was no significant difference in the measured direct [O_2_] and OCR over the 10‐day storage for PCs stored at 100% or 50% [PLT] (Figure 5A,B, respectively). A significant decrease in [O_2_] was observed in PCs stored at 100% [PLT] compared with PCs stored at 50% [PLT] (day 2; *p* < .001, day 4; *p* < .01, day 6; *p* < .001, day 8; *p* < .01, day 10; *p* < .05) (Figure 5A). There was no significant difference in the OCR between PCs stored at 100% and 50% [PLT], when normalized for PLT count.

### 5.6 Measures of PC function and quality control

**FIGURE 6:**
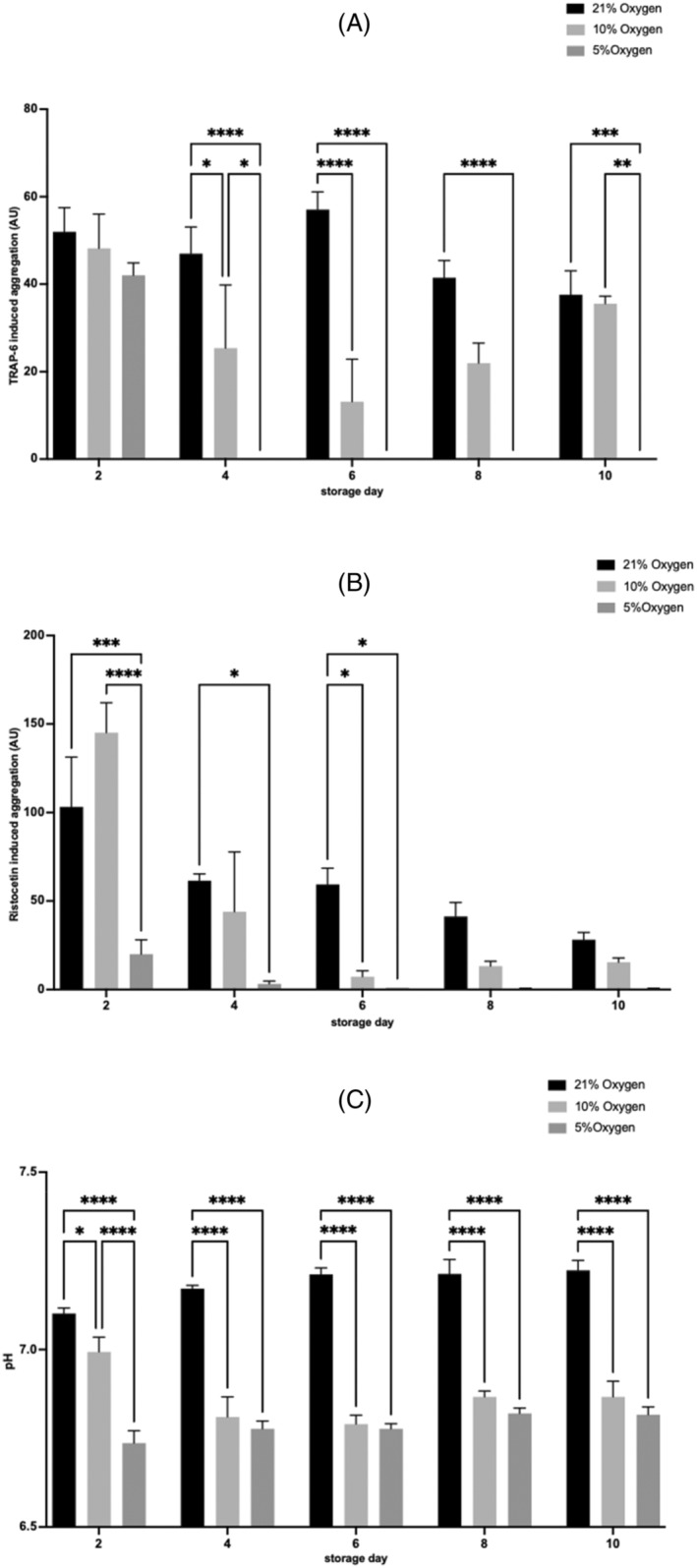
Platelet concentrate (PC) function and quality control measures over 10 days of storage. (A) Platelet (PLT) aggregation responses to TRAP‐6. (B) PLT aggregation responses to Ristocetin. (C) pH of PCs. Error bars denote SEM. Significance was assessed by two‐way ANOVA following by a Bonferroni test and graphs are denoted as follows: * = *p* < .05, ** = *p* < .01, *** = *p* < .001, **** = *p* < .0001. *n* = 3 (*n* = 6 for control).

TRAP‐6 induced aggregation was reduced for PCs stored at an external O_2_ of 5% [O_2_] versus 21% [O_2_] from day 4 onward (day 4, *p* < .0001; day 6, *p* < .0001; day 8, *p* < .0001; day 10, *p* < .001) (Figure 6A). A significantly lower TRAP‐6 response was also reported at 10% [O_2_] compared with 21% [O_2_] on day 4 (*p* < .05) and day 6 (*p* < .0001). A significantly lower TRAP‐6 responsiveness was reported on day 4, and day 10 at 5% [O_2_] compared with 10% [O_2_] (day 4, *p* < .05; day 10, *p* < .01).

Ristocetin induced aggregation of PC stored at 5% [O_2_] was significantly reduced on days 2 to 6 compared with 21% [O_2_] (day 2, *p* < .001; day 4, *p* < .05; day 6, *p* < .05), and on day 2 and 6 compared with 10% [O_2_] (day 2, *p* < .0001; day 6, *p* < .05) (Figure 6B).

The pH of PC stored at 5% [O_2_] was significantly lower from day 2 onward compared with 21% [O_2_] (day 2, *p* < .0001; day 4, *p* < .0001; day 6, *p* < .0001; day 8, *p* < .0001; day 10, *p* < .0001), and 10% [O_2_] (day 2, *p* < .05; day 4, *p* < .0001; day 6, *p* < .0001; day 8, *p* < .0001; day 10, *p* < .0001, Figure 6C).

### 5.7 PC function and quality control measures at reduced [PLT]

**FIGURE 7:**
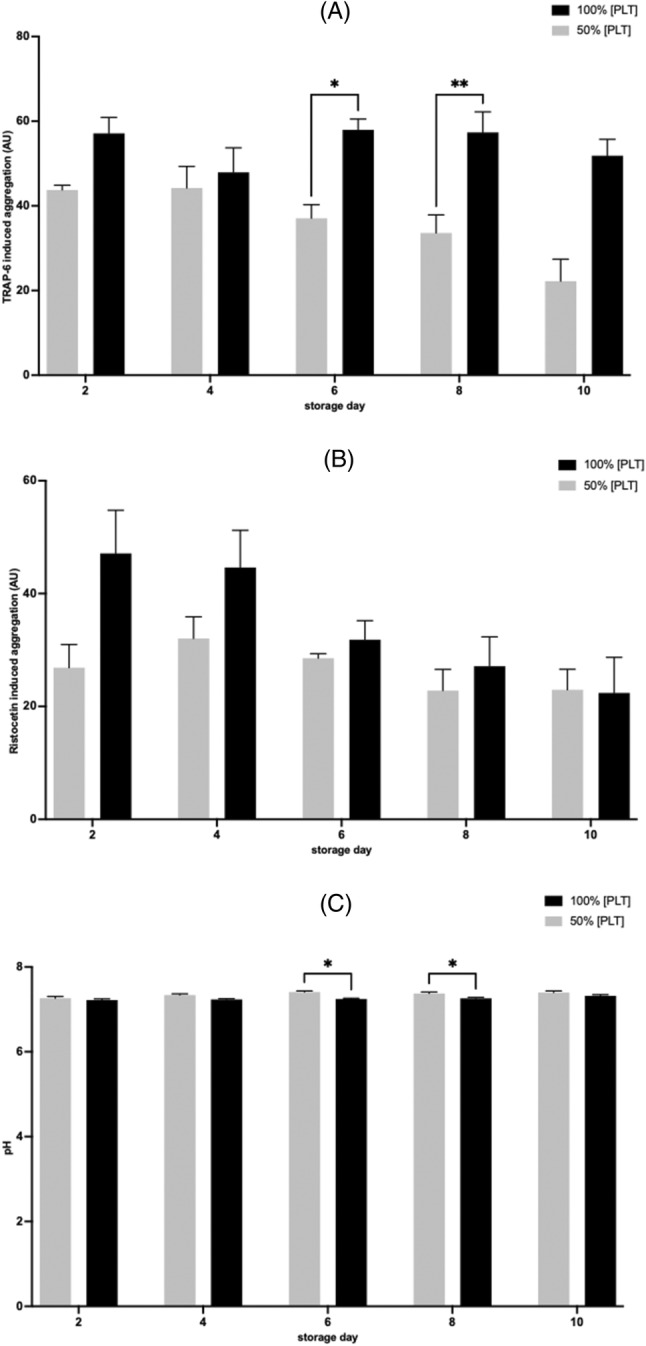
Platelet concentrate (PC) function and quality control measures over 10 days storage when reducing [PLT]. (A) Platelet (PLT) aggregation responses to TRAP‐6. (B) PLT aggregation responses to Ristocetin. (C) pH of PC units over time at 100% [PLT] and 50% [PLT]. Error bars denote SEM. Significance was assessed by two‐way ANOVA followed by a Bonferroni test and denoted as follows: * = *p* < .05, ** = *p* < .01, *** = *p* < .001, **** = *p* < .0001, *n* = 4.

TRAP‐6 induced aggregation was not significantly different in PCs stored at 50% [PLT] compared with the 100% [PLT] on day 2, day 4, and day 10 of storage. However, TRAP‐6 induced aggregation significantly decreased on day 6 (*p* < .05), and day 8 (*p* < .01) (Figure 7A). Ristocetin induced aggregation in PC stored at 50% [PLT] was not significantly different on days 2, 4, 6, 8, and 10 when compared with 100% [PLT] (Figure 7B).

The pH did not fall below the 6.4 QC threshold value adopted by blood service establishments throughout storage at any condition. On day 6 and 8, the pH in 50% [PLT] is significantly higher compared with 100% [PLT] (day 6, *p* < .05; day 8, *p* < .05). Over storage, pH did not change for 100% [PLT] PC (7.2 ± 0.05 vs. 7.32 ± 0.05, *p* = .0538). However, the 50% [PLT] PC showed a significant increase in pH over the 10‐day storage period (7.2 ± 0.05 vs. 7.4 ± 0.07, *p* = .0112) (Figure 7C).

## 6 DISCUSSION

Storage of human‐derived cells under conditions which closely mimic the “physiological‐like” O_2_ has been reported to increase speed to confluence and decrease metabolic stress, resulting in improved cell viability and maintenance of characteristic function.^11^ This study investigated whether ambient [O_2_] or [PLT] could be optimized during PC storage and improve PLT quality or lifetime. EPR oximetry was applied to make direct measures of [O_2_] and OCR within PC. The results show O_2_ availability and function is negatively affected by reducing the ambient [O_2_] but O_2_ availability is enhanced by reducing the stored [PLT].

Though O_2_ availability within the PC unit at an ambient 10% [O_2_] is reduced, the OCR remained the same as that exhibited by PC stored at 21% [O_2_] and it was noticeably similar to the OCR exhibited by fresh PRP. This suggests the O_2_ supply exceeds that of the O_2_ demand per unit volume. However, at an ambient 5% [O_2_], the measured internal [O_2_] and OCR both decreased to close to zero. Since mitochondrial respiration should proceed at near maximal rate at [O_2_] above 0.08 μM,^25^ this suggests the O_2_ demand at 5% [O_2_] was greater than the O_2_ supply per unit volume. Under this condition, our model predicts that at least 60% of the PLTs within the unit experience complete anoxia. Alternatively, reducing the [PLT] by 50% at an ambient 21% [O_2_] resulted in increased internal [O_2_] without affecting OCR (when normalized to [PLT]). Moreover, the O_2_ distribution throughout the PC stored at 50% [PLT] was maintained closer to homogeneity compared with the PC stored at 100% [PLT]. This is consistent with the findings by Torres et al., indicating [PLT] has the biggest effect on internal partial pressure of oxygen.^26^ Consistent with this, the [O_2_] measured in PAS only samples (without PLTs consuming O_2_) closely reflected the ambient O_2_ under each experimental condition.

The reported pH values in all conditions were maintained within JPAC guidelines of pH ≥6.4 (Figure 4C).^27^ PC used within this study were stored in a 65:35 ratio of PAS to plasma, which has recently been questioned in terms of accuracy of pH to reflect PC quality.^28^ Bicarbonate in plasma, and to a lesser extent, the acetate and phosphate in PAS, can buffer pH and reduce the likelihood of pH falling below 6.4. PC stored at 5% [O_2_] would likely rapidly exhaust glucose stores and produce large amounts of lactate, limiting ATP production and the PLTs ability to perform energetically demanding processes.^29^, ^30^ Although ATP might contribute to the lack of function observed, it cannot completely explain our finding as PC stored at 10% [O_2_] maintained a maximal OCR while showing reduced functionality, as measured by agonist‐induced aggregation.

PLT activation during PC storage is likely the cause of reduced function when testing responses to an exogenous agonist. Although PLT activation was not directly measured, [O_2_] has been thought to contribute to hypoxia‐induced, PLT activation. PLT hyperactivity has been reported in numerous oxygen compromising conditions, (e.g., high altitude, chronic obstructive pulmonary disease [COPD], and ischemia), where the enhanced PLT activity gives rise to increased thrombotic risk.^31^, ^32^, ^33^, ^34^ Increased PLT activation during storage can affect the hemostatic potential of the PC in a few ways. First, PLT activation reduces the proportion of the overall PLT population within the PC unit available to respond to secondary challenge by an agonist, thereby reducing the aggregation response observed in vitro. This agrees with Schoenfeld et al., who measured CD62P surface expression to reflect PLT activation during storage and observed reduced response to subsequent ADP challenge.^35^ Second, PLT activation has also been proposed to effect receptor shedding, with receptors for thrombin (PAR1, PAR4), collagen (GPVI), and von Willebrand factor (CD42b) shed during PC storage.^36^, ^37^ Finally, increased activation during storage is implicated in platelet clearance processes by leucocytes with a subsequent reduction in component efficacy posttransfusion.^38^

Multiplate aggregometry was used to infer PLT aggregation in response to a given agonist in vitro, but the authors acknowledge this assay primarily reflects the extent of primary hemostasis. It is possible other clotting parameters may be influenced by altered storage [O_2_] and [PLT]. Moreover, reports have demonstrated PLT aggregation recovery in vivo.^39^, ^40^ The clinical significance of our findings must be considered in this light and further studies should look to address this.

The authors acknowledge a number of limitations with data modeling. Agitation was applied throughout PLT storage in accordance with standardized conditions as it is thought to negate the development of regions of local anoxia throughout the PC by ensuring a sufficient PLT suspension is maintained.^6^ The mathematical model utilized in this study was therefore based on the assumption of homogenous mixing of PLTs throughout the PC, and therefore does not consider the possibility of boundary effects or local anoxia induced by regions of denser PLT populations. However, since the [PLT] per parallelepiped is constant, any local variations in OCR across the PC must collectively account for the overall OCR of the parallelepiped, we thus reasoned the [O_2_] at the bag midpoint will likely be the same in a homogenous and heterogenous suspension in this model. Moreover, with the O_2_ supply being far greater than O_2_ demand per unit volume at both 21% and 10% [O_2_], together with a predicted near homogenous O_2_ distribution, it is likely local anoxia does not exist under standard blood banking conditions.

Furthermore, the model assumes the PC bag itself can be considered a cuboidal shape allowing each “parallelepiped” to be identical; however, in practice, the PLT bag has a slight curvature, suggesting areas further from the bag center may have altered diffusive characteristics unrepresented in this study. The study is also limited to a low sample number. This was addressed partly by utilizing a pool and split study design^27^ which provided some statistical benefits to the study, including limiting the inter‐donor variability, and an opportunity to conduct pairwise analysis through splitting the sample PC.

This study concludes O_2_ is not limiting under standard blood banking conditions. However, reducing the storage [O_2_] to reflect physiological levels appears to significantly alter O_2_ distribution within the PC and negatively impacts PLT functionality and quality, and therefore is not a viable storage option. Reducing [PLT] however improves O_2_ availability and maintains functionality comparable to 100% [PLT]. The latter implies that reducing PC [PLT] may be advantageous in maintaining patient outcomes and could explain why clinical studies investigating reduced [PLT] transfusion have shown no significant risk in bleeding times in transfused patients.^41^, ^42^, ^43^ Evidence has suggested that patients require ~7100 PLT/μL/day to maintain hemostasis,^44^ but this is highly dependent on the quality of the PLTs. The European Directory for the Quality of Medicines and Healthcare (EDQM) Guide stated a minimum of 2 × 10^11^ PLTs per pool, and the mean PLT content in a “standard” pool to approximate to 3.0–3.5 × 10^11^,^45^ there is, however, international variation with the UK specifications indicating >2.40 × 10^11^/pool.^46^ The appropriate “dose” of therapeutic platelets has been assessed in randomized controlled trials and following a systematic review of regularly transfused, therapy induced hypoproliferative thrombocytopenia patients the effectiveness of a lower dosage of platelets was concluded.^43^ In this study those receiving low dose platelets achieved equal hemostatic control to those receiving standard dose platelets although intervals between platelet transfusions may decrease. The results of the current study raises the possibility that transfusion of PC with reduced [PLT], exhibiting similar in vitro functionality and hemostatic quality, can achieve the same clinical efficacy as the current recommended concentration.^47^ It should be emphasized that PCs stored in alternative additive solutions or PCs undergoing pathogen reduction treatment may yield different outcomes. Therefore, further investigations with lower platelet doses are required to fully understand these variations.

If translated into practice, our findings could have potential to improve the availability of blood bank stocks in that four whole blood donations could yield two PC units (as opposed to one PC unit presently). However, future research should first assess if a reduction in [PLT] within a PC unit is clinically appropriate and in which clinical context this would be appropriate. Given that reduction of [PLT] improved O_2_ homogeneity and upholds PLT aggregation throughout storage investigated herein, our results suggest the potential to improve blood bank stocks and/or reduce the donations required to maintain PC supply while upholding therapeutic quality.

## CONFLICT OF INTEREST STATEMENT

The authors have disclosed no conflicts of interest.

## Supporting information


**Data S1.** Supporting Information.
