# Supplementary material for: Enhanced oxygen availability and preserved aggregative function in platelet concentrates stored at reduced platelet concentration
Source: Transfusion. 2024 Dec 14;65(3):575–87. doi: 10.1111/trf.18101 (PMC11925141; doi:10.1111/trf.18101)
Supplement: Supplementary file 1 — Data S1. Supporting Information. [file TRF-65-575-s001.docx]

**Supplement**

**A**

**B**

**Supplement Figure 1 -** **PDT signal broadening in the presence of oxygen. (A)** Exemplar EPR spectra recorded in nitrogen and in 21% O_2_ showing spectral linewidth broadening. (B) Calibration of linewidth against [O_2_] equilibrated in N_2_, 5%, 10% and 21% O_2_.(R^2^=0.9992).

**Supplement Figure 2 – Measurement of OCR by EPR Oximetry.** A fixed concentration and volume of PLTs are drawn into a quartz glass capillary sealed at both ends. EPR spectra were recorded every 60 seconds and the rate of linewidth change over time taken to reflect the change in O_2_ with time, calculated from a standard curve.

EPR Spectrometer Conditions.

Spectra were recorded on a Bruker ER 220DSRC ESR spectrometer. The sample was placed into the EPR cavity and maintained at 22^o^C throughout (this maintained the storage conditions of PC)

Typical spectrometer conditions applied were –

Modulation Amplitude – 0.07Gauss

Modulation Frequency – 86kHz

Attenuator – 30dB (Power = 0.5mW)

Centre Field – 3472.4G

Scan width – 2G

**Supplement Table 1 – Characteristics of PC samples utilised in these studies**

| **Parameter**  **(unit)** | **Mean ± s.d.** |
| --- | --- |
| Platelet count  (x 10^9^/L) | 1190 ± 88 |
| MPV (Mean Platelet Volume)  (fL) | 8.35 ± 0.14 |
| RBC (residual)  (x 10^12^/L) | 0.04 ± 0.008 |
| Hb free  (g/L) | <lower limit cut off for PC |
| WBC count | <lower limit cut off for PC |

Samples in dry EDTA were mixed for 30 minutes and assayed on a Pentra XL80 haematology analyser (HoribaABX Diagnostics, Montpellier, France).

Residual white cell counts were measured on a Beckman Coulter DXFlex flow cytometer, using the company’s DNA-Prep kit (Beckman Coulter, Miami, FL). A single platform absolute cell counting protocol was used, with a three-minute acquisition per sample
